# Resveratrol-enhanced autophagic flux ameliorates myocardial oxidative stress injury in diabetic mice

**DOI:** 10.1111/jcmm.12312

**Published:** 2014-06-01

**Authors:** Bo Wang, Qing Yang, Yuan-yuan Sun, Yi-fan Xing, Ying-bin Wang, Xiao-ting Lu, Wen-wu Bai, Xiao-qiong Liu, Yu-xia Zhao

**Affiliations:** a Department of Traditional Chinese Medicine, Qilu Hospital of Shandong UniversityJinan, China; b Institute of Pathogen Biology, Shandong UniversityJinan, China; c Key Laboratory of Cardiovascular Remodeling and Function Research, Qilu Hospital of Shandong UniversityJinan, China

**Keywords:** cardiomyocyte, diabetes, autophagy, oxidative stress

## Abstract

Autophagic dysfunction is observed in diabetes mellitus. Resveratrol has a beneficial effect on diabetic cardiomyopathy. Whether the resveratrol-induced improvement in cardiac function in diabetes is *via* regulating autophagy remains unclear. We investigated the mechanisms underlying resveratrol-mediated protection against heart failure in diabetic mice, with a focus on the role of sirtuin 1 (SIRT1) in regulating autophagic flux. Diabetic cardiomyopathy in mice was induced by streptozotocin (STZ). Long-term resveratrol treatment improved cardiac function, ameliorated oxidative injury and reduced apoptosis in the diabetic mouse heart. Western blot analysis revealed that resveratrol decreased p62 protein expression and promoted SIRT1 activity and Rab7 expression. Inhibiting autophagic flux with bafilomycin A1 increased diabetic mouse mortality and attenuated resveratrol-induced down-regulation of p62, but not SIRT1 activity or Rab7 expression in diabetic mouse hearts. In cultured H9C2 cells, redundant or overactive H_2_O_2_ increased p62 and cleaved caspase 3 expression as well as acetylated forkhead box protein O1 (FOXO1) and inhibited SIRT1 expression. Sirtinol, SIRT1 and Rab7 siRNA impaired the resveratrol amelioration of dysfunctional autophagic flux and reduced apoptosis under oxidative conditions. Furthermore, resveratrol enhanced FOXO1 DNA binding at the Rab7 promoter region through a SIRT1-dependent pathway. These results highlight the role of the SIRT1/FOXO1/Rab7 axis in the effect of resveratrol on autophagic flux *in vivo* and *in vitro*, which suggests a therapeutic strategy for diabetic cardiomyopathy.

## Introduction

Diabetes mellitus is a well-recognized risk factor for heart failure. Many studies have suggested that type 1 and type 2 diabetes exhibit systolic and diastolic dysfunction, LV hypertrophy and fibrosis [1,2]. Increased reactive oxygen species (ROS) generation and impaired ROS clearance contribute to the development and progression of diabetic cardiomyopathy [3]. Accumulated ROS that arises from mitochondria has been shown in cardiomyocytes that are exposed to hyperglycaemia [4]. Hyperglycaemia may produce ROS through the formation of advanced glycation end products and activation of NADPH oxidase *via* protein kinase C [5]. Evidence also exists for increased production of ROS from reduced activity of neuronal nitric oxide synthase (nNOS) coupled with increased activation of xanthine oxidoreductase [6].

Autophagy occurs at a basal rate in most cells, eliminating protein aggregates and damaged organelles such as mitochondria to maintain homoeostasis. Recent studies showed deficient autophagy in diabetic heart [7]. Impaired autophagy induced by autophagy-associated gene (ATG) 5 knockout results in increased dysfunctional mitochondria and accumulation of ROS [8]. Additionally, excessive ROS levels lead to dysfunction of autophagic activities and apoptosis [9]. A cross-talk exists between autophagy and oxidative stress. The exact mechanisms are largely unknown, especially in diabetic cardiomyopathy.

Resveratrol is a natural polyphenol found in peanuts, grapes and red wine [10]. It is shown to attenuate cardiomyocyte apoptosis in heart failure and improve cardiac function in diabetes through SIRT1-dependent way [11,12]. Recent studies suggest that resveratrol may induce cardiac autophagy after hypoxia-reoxygenation or ischemia–reperfusion [13]. However, whether resveratrol can regulate autophagy in diabetic cardiomyopathy has not been evaluated.

In the present study, we hypothesized that resveratrol might have a protective effect by improving impaired autophagic function in diabetic cardiomyopathy. We investigated the effect of resveratrol on autophagy in hearts of mice with diabetes induced by streptozotocin (STZ) and in cultured H9C2 cells. The role of SIRT1 in resveratrol-mediated regulation of autophagy was identified *in vivo* and *in vitro*.

## Materials and methods

### Animals and experimental protocols

All animal protocols were approved by the Shandong University Animal Care Committee, and procedures were carried out in accordance with the Guide for the Care and Use of Laboratory Animals published by the National Institutes of Health (Documentation 55, 2001). Wild-type male C57BL/6J mice 12 weeks old were used for experiments. Diabetes was induced by intraperitoneal injection of STZ (Sigma-Aldrich, St. Louis, MO, USA), 50 mg/kg, for 5 consecutive days. After 7 days, non-fasted blood glucose levels >20 mmol/l indicated diabetes [14].

Mice were randomly divided into seven groups (*n* = 15 in each group) for treatment: Control, STZ, STZ+low-dose resveratrol (Sigma-Aldrich; STZ+RL), STZ+high-dose resveratrol (STZ+RH), STZ+RH+bafilomycin A1 (Sigma-Aldrich; STZ+RH+B), Control+ bafilomycin A1 (Control+B) and STZ+ bafilomycin A1 (STZ+B). Control, STZ, Control+B and STZ+B mice were fed a regular diet; STZ+RL mice were fed a diet enriched with 0.06% resveratrol (about 60 mg/kg/day); STZ+RH and STZ+RH+B mice were fed a diet enriched with 0.3% resveratrol (about 300 mg/kg/day). At the end of 12 weeks, STZ+RH+B, Control+B and STZ+B mice were intraperitoneally treated with bafilomycin A1 (0.3 mg/kg) daily for 4 weeks. This dose of bafilomycin A1 used here has been previously reported as successfully suppressing autophagy, without apparent adverse effects [15]. The other four groups were injected with vehicle (Dimethyl Sulfoxide). Details are given in Data S1 online.

### Echocardiography

Transthoracic echocardiography involved use of the Vevo 770 imaging system equipped with 30-MHz transducer (VisualSonics, Toronto, ON, Canada). Mice were anaesthetized with a mixture of isoflurane (2%) and O_2_ (2 l/min.). M-mode echocardiography and pulsed-wave Doppler echocardiography of mitral inflow were performed as described previously [16]. Details are given in Data S1 online.

### Preparation of heart tissue samples

At the end of 16 weeks, mice were anaesthetized with ketamine (20 mg/kg) and xylazine (1 mg/kg) until they were not responsive to toe pinching, then the hearts were harvested for weighting, histological and biochemical assays.

### Transmission electron microscopy (TEM)

Heart tissues were processed for TEM assay according to routine procedures. Autophagosomes with a double membrane in cardiomyocytes were observed by use of an H-7000FA TEM (Hitachi, Tokyo, Japan). The number of autophagosomes was calculated from a random selection of eight fields in each sample. Details are given in Data S1 online.

### Real-time RT-PCR

Total RNA was extracted from heart tissue by use of TRIzol reagent (Invitrogen, Carlsbad, CA, USA) and reverse transcribed by use of a cDNA reverse transcription kit (Takara Biotechnology, Tokyo, Japan). The sequences of primers are listed in Table S1. The mRNA levels were calculated on the basis of threshold cycle (CT) values. The mRNA expression of target genes was normalized to that of β-actin as the control by the 2^−ΔΔCT^ method. Each experiment was repeated in triplicate.

### Histology, immunohistochemistry, and TUNEL analysis

Myocardial sections were stained with haematoxylin and eosin, or Masson's trichrome. Digital images were obtained at magnification 400× by microscopy (Olympus, Tokyo, Japan). Single cardiomyocyte with nucleus was chosen from haematoxylin and eosin-stained transverse sections. Forty chosen myocytes were quantified to assess the mean of cross-sectional area by use of Image-Pro Plus 5.0 (Media Cybernatics, Houston, TX, USA). For fibrosis degree, 10 randomly chosen frames from Masson's trichrome-stained sections were analysed with Image-Pro Plus 5.0.

Primary antibody against 8-OHdG (Abcam, Cambridge, UK) was used for immunohistochemistry. Paraffin-embedded sections underwent TUNEL staining of nuclei positive for DNA strand breaks by use of an apoptosis detection kit (Millipore, Billerica, MA, USA). The 8-OHdG- or TUNEL-positive cells were counted manually under 400× magnification within LV; 10 microscopic fields were analysed randomly from each sample.

### Malondialdehyde (MDA) and 15-F_2t_-isoprostane (15-F_2t_-IsoP) content assay

Malondialdehyde content in heart tissues was measured by use of a kit (Jiancheng Bioengineering Institute, Nanjing, China). 15-F_2t_-IsoP in heart tissue homogenate was measured by using an enzyme immunoassay kit (Cayman Chemical, Ann Arbor, MI, USA) as previously described [17]. Each experiment was carried out in triplicate.

### Cell culture and treatment

H9C2 cell lines (ATCC, Manassas, VA, USA) were cultured in low-glucose DMEM (Invitrogen) supplemented with 10% foetal bovine serum (Invitrogen), 100 U/ml penicillin and 100 μg/ml streptomycin (Invitrogen). On reaching 80% confluence, cells were treated with 0, 100, 200 or 400 μM H_2_O_2_ for 3, 6 or 12 hrs. An amount of 100 nM bafilomycin A1 (Sigma-Aldrich) was used to inhibit autophagic flux. In some experiments, cells were treated with 400 μM H_2_O_2_ with or without 25 μM resveratrol or 25 μM sirtinol (Sigma-Aldrich) for 12 hrs.

### Cellular ROS detection

Celluar ROS production was determined by fluorescence microspectrometry with chemical probes that become fluorescent on reaction with ROS. The cell-permeant 2′,7′- dichlorodihydrofluorescein diacetate (H_2_DCFDA; Molecular Probes, Carlsbad, CA, USA) was used to detect cellular oxidants as previously described [18]. The fluorescence intensities were measured with a microplate reader (Varioskan Flash, Thermo Fisher, Germany). Fluorescence images were obtained by fluorescent microscopy (Olympus). Details are given in Data S1 online.

### GFP-LC3 lentivirus transfection

Lentiviral vectors carrying GFP and LC3 cDNA and control vectors were constructed by Genechem (Shanghai, China). H9C2 cells were transfected with lentiviral vectors. After 72 hrs, cells were exposed to 100 or 400 μM H_2_O_2_ for 12 hrs with or without 100 nM bafilomycin A1. Fluorescence images were obtained by fluorescent microscopy (Olympus). Autophagosomes from a random selection of 20 cells were calculated as described [19].

### Small interference RNA (siRNA) transfection

H9C2 cells were transiently transfected with SIRT1, Rab7 or control siRNA (GenePharma, Shanghai, China) by use of Lipofectamine 2000 (Invitrogen). The siRNA sequences were for SIRT1, sense, 5′-CACCUGAGUUGGAUGAUAUTT-3′, and antisense, 5′-AUAUCAUCCAACUCAGGUGTT-3′; and Rab7, sense, 5′-UACUGGUUCAUGAGCGAUGUCUUUC-3′, and antisense, 5′-GAAAGACAUCGCUCAUGAACCAGUA-3′. Cells were treated with resveratrol or H_2_O_2_ after siRNA transfection.

### Western blot analysis

Total protein from heart tissues or cells was extracted by use of RIPA Lysing Buffer (Beyotime, Shanghai, USA). Protein was incubated with the primary antibodies anti-LC3, anti-beclin1, anti-acetyl-histone 3 (Lys9), anti-histone 3, anti-β-actin (Cell Signaling Technology, Danvers, MA, USA); anti-SIRT1, anti-FOXO1, anti-cleaved caspase 3 (Abcam); anti-acetyl-FOXO1 (Lys259, Lys262, Lys271; Santa Cruz Biotechnology, Santa Cruz, CA, USA); anti-p62 and anti-Rab7 (Sigma-Aldrich), then horseradish peroxidase-conjugated AffiniPure goat anti-rabbit and antimouse IgG secondary antibodies. Bands were revealed by use of the FluorChem E data system (Cell Biosciences, Santa Clara, CA, USA) and quantified by densitometry with Quantity One 4.52 (Bio-Rad, Hercules, CA, USA). Each assay was repeated at least three times.

### Electromobility shift assay (EMSA)

Nuclear proteins were extracted from H9C2 cells with or without SIRT1 siRNA transfection, resveratrol and H_2_O_2_ treatment by using Nuclear Extract kit (Viagene Biotech, Beijing, China). Nuclear FOXO1 protein was captured by using 5′-biotinylated FOXO1 binding site oligo sequence 5′-ACTTAATGGTTTGTTTACTTTCTTA A-3′ and subjected to DNA binding activity by using non-radioactive EMSA kit (Pierce, Rockford, IL, USA) as described previously [20]. Specificity was demonstrated by adding 100-fold excess cold unlabeled oligonucleotide. To achieve a supershift, anti-FOXO1 antibody (Abcam) was incubated with nuclear extract before EMSA. The membrane was cross-linked and visualized by using FluorChem E data system (Cell Biosciences). Each assay was performed in triplicate. Details are given in Data S1 online.

### Statistical analysis

Data are presented as mean ± SEM. Differences between groups were compared by one-way anova. Survival analysis involved the Kaplan–Meier method, and between-group differences in survival were tested by the Log-rank (Mantel-Cox) test. SPSS v16.0 (SPSS Inc., Chicago, IL, USA) was used for analysis. *P* < 0.05 was considered statistically significant.

## Results

### Resveratrol ameliorates deficient myocardium autophagic flux in diabetic mice and attenuates mouse mortality

**Fig. 1:**
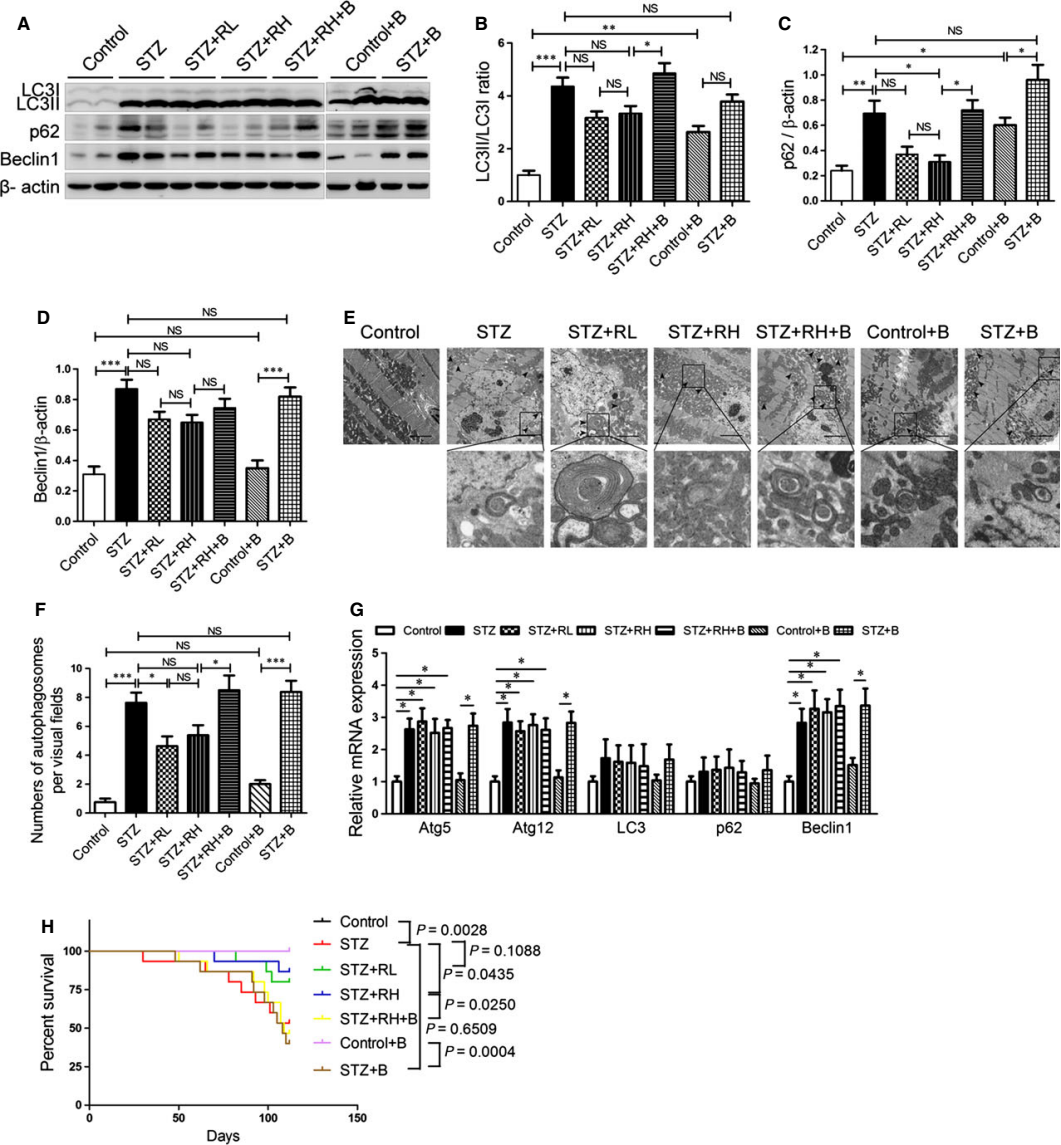
Resveratrol promotes autophagic flux in diabetic mouse hearts and attenuates mouse mortality. (**A**) Western blot analysis of autophagy-related proteins: LC3I, LC3II, p62, and beclin1. (**B**–**D**) Relative expression of LC3II to LC3I ratio (**B**), p62 (**C**) and beclin1 (**D**) in mouse groups (*n* = 4). (**E**) Electron micrographs of cardiomyocytes in different groups (5000×). Black arrowheads indicate autophagosomes; scale bar, 2 μm. (**F**) Quantification of autophagosomes by transmission electron microscope (5000×; *n* = 5). (**G**) RT-PCR analysis of relative mRNA levels of autophagy-related genes in different mouse groups (*n* = 4). (**H**) Kaplan-Meier survival curves for different mouse groups (*n* = 15). Data are mean ± SEM. *P < 0.05, **P < 0.01, ***P < 0.001.

The ratio of LC3II to LC3I was greater in diabetic than control mouse hearts and was significantly higher after bafilomycin A1 treatment to inhibit autophagic flux (Fig. 1A and B). As compared with controls, diabetic hearts showed increased p62 expression, which was markedly inhibited by resveratrol treatment (Fig. 1A and C). Bafilomycin A1 treatment reversed the effect of resveratrol on p62 expression (Fig. 1A and C). Moreover, bafilomycin A1 significantly increased p62 expression in hearts of control mice, but not in hearts of STZ mice (Fig. 1A and C). The protein level of beclin1 was higher in all diabetic than in control hearts (Fig. 1A and D). Neither Resveratrol nor bafilomycin A1 had significant effect on beclin1 expression (Fig. 1A and D).

TEM assay confirmed the significant accumulation of autophagosomes with double-membraned structures in diabetic mouse hearts alone and diabetic hearts with bafilomycin A1 (Fig. 1E and F). Resveratrol decreased the number of autophagosomes in diabetic hearts, which was reversed by bafilomycin A1 treatment (Fig. 1E and F). Interestingly, almost all of the autophagic substrates detected in diabetic hearts were mitochondria (Fig. 1E).

ATG5, ATG12 and Beclin1 (ATG6) mRNA expression was enhanced in all diabetic mouse groups (Fig. 1G). However, compared with controls, diabetic hearts showed no significant changes in LC3 (ATG8) and p62 mRNA expression (Fig. 1G).

High-dose resveratrol treatment reduced STZ-induced mortality in mice. The mortality was increased with bafilomycin A1 treatment for 4 weeks in diabetic mice, but not in control mice (Fig. 1H). These results suggest defective autophagic flux in diabetic hearts, which could be ameliorated by resveratrol. Inhibition of autophagic flux may increase the mortality of diabetic mice.

### Resveratrol improved cardiac function and alleviated hypertrophy and fibrosis in diabetic mouse hearts depending on the regulation of autophagic flux

**Fig. 2:**
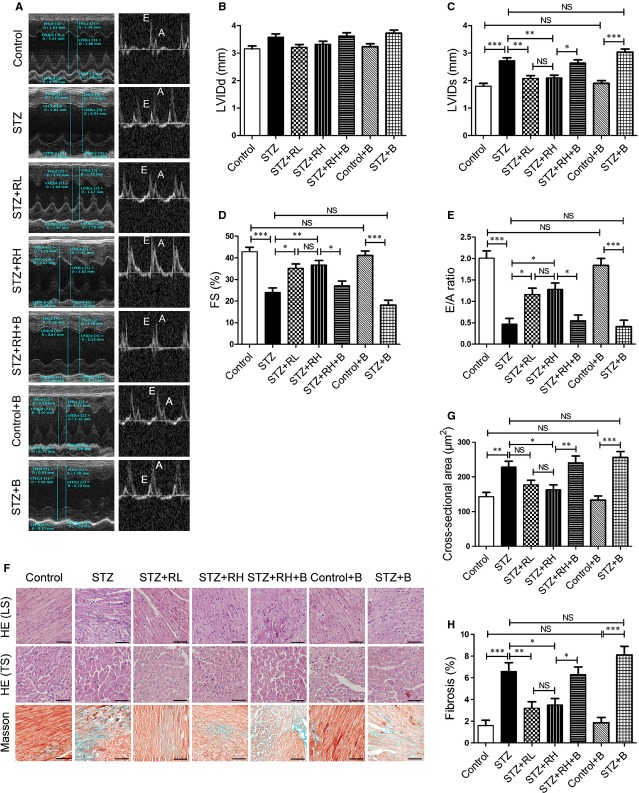
Resveratrol ameliorates cardiac dysfunction, hypertrophy and fibrosis depending on the regulation of autophagic flux. (**A**) Transthoracic echocardiography in mouse groups at the end of 16 weeks. (**B**–**E**) Evaluations of LV internal dimension at diastole (LVIDd) (**B**), LV internal dimension at systole (LVIDs) (**C**), fractional shortening (FS%) (**D**) and peak E to peak A ratio (E/A) (**E**). (**F**) haematoxylin and eosin-stained longitudinal sections (LS), transverse sections (TS) and Masson's trichrome-stained sections of mouse left ventricles; scale bar, 50 μm. (**G**) Quantification of cross-sectional area of cardiomyocytes from haematoxylin and eosin-stained sections. (**H**) Quantification of cardiac fibrosis area from Masson's trichrome-stained sections. Data are mean ± SEM. *n* = 5. *P < 0.05, **P < 0.01, ***P < 0.001.

We further investigated whether resveratrol could ameliorate cardiomyopathy in diabetic mice. In general characteristics analysis, resveratrol had no significant effect on the levels of blood glucose (Table S2). It was found to inhibit the body weight loss and heart weight increase in diabetic mice, but the differences were not statistically significant (Table S2). To evaluate the systolic and diastolic function of hearts, we found that after 16 weeks' treatment, resveratrol reduced LVIDs values and prevented the decrease in EF%, FS% and E/A in diabetic hearts, although LVIDd values did not show significant difference among groups. The effect of resveratrol on cardiac function was attenuated by bafilomycin A1 treatment (Fig. 2A–E, Table S3). However, bafilomycin A1 had no significant effect on cardiac function of controls (Fig. 2A–E, Table S3).

Pathology revealed that resveratrol decreased the cross-sectional area of cardiomyocytes (Fig. 2F and G) and ratio of fibrosis (Fig. 2F and H) in diabetic mice. Compared with resveratrol, bafilomycin A1 aggravated the hypertrophy and the fibrosis degree in hearts from diabetic mice (Fig. 2F and H). These findings suggest that inhibition of autophagic flux abolishes the protective effects of resveratrol on cardiac function, hypertrophy and fibrosis in diabetic mice.

### Resveratrol ameliorates oxidative stress and apoptosis in diabetic mouse hearts depending on regulation of autophagic flux

**Fig. 3:**
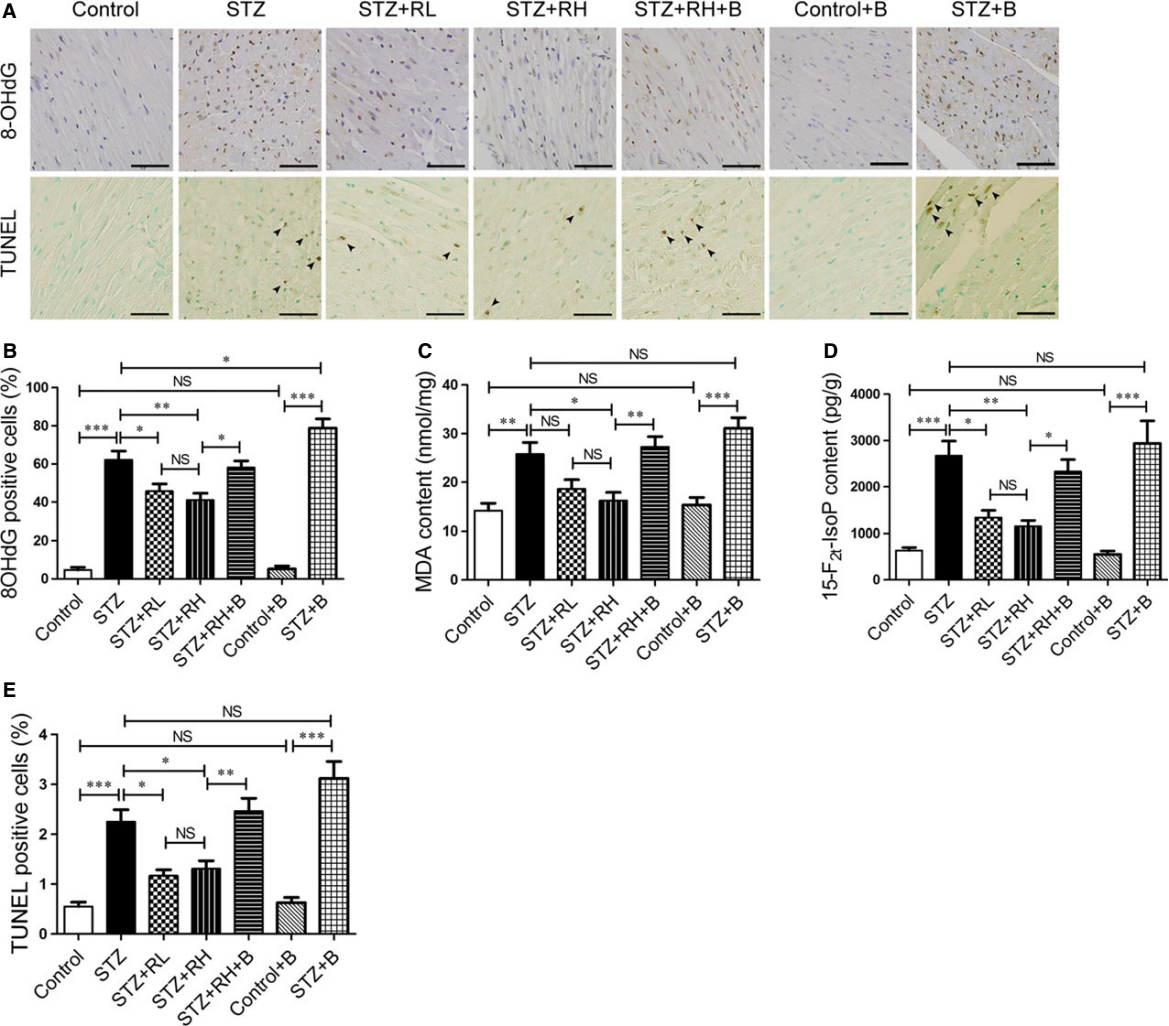
Resveratrol attenuates oxidative stress and apoptosis in diabetic hearts depending on regulation of autophagic flux. (**A**) 8-OHdG-immunostained and TUNEL-stained sections. Black arrowheads indicate TUNEL-positive nuclei (brown). Scale bar, 50 μm. (**B**) Quantitative analysis of the proportion of 8-OHdG-positive nuclei. (**C**) Quantification of malondialdehyde (MDA) level in homogenized fresh heart tissues. (**D**) Quantification of 15-F_2t_-isoprostane (15-F_2t_-IsoP) content in homogenized heart tissues. (**E**) Quantitative analysis of the proportion of TUNEL-positive nuclei. Data are mean ± SEM. *n* = 4. *P < 0.05, **P < 0.01, ***P < 0.001.

The 8-OHdG, MDA and 15-F_2t_-IsoP are markers of oxidative damage *in vivo*. Diabetic hearts showed a significant accumulation of 8-OHdG-positive cells, which was attenuated by resveratrol treatment (Fig. 3A and B). Inhibition of autophagic flux with bafilomycin A1 increased the expression of 8-OHdG in diabetic heart, but not in control (Fig. 3A and B). Resveratrol decreased the content of MDA and 15-F_2t_-IsoP in diabetic hearts; however, the content was increased by bafilomycin A1 (Fig. 3C and D). TUNEL assay revealed an increased ratio of TUNEL-positive cells in diabetic mouse hearts, which was reduced with resveratrol treatment, but reversed with bafilomycin A1 treatment (Fig. 3A and E). Thus, inhibition of autophagic flux abolished the protective effects of resveratrol on oxidative damage and apoptosis in diabetic mouse hearts.

### Resveratrol activates SIRT1 and enhances Rab7 expression in diabetic mouse hearts

**Fig. 4:**
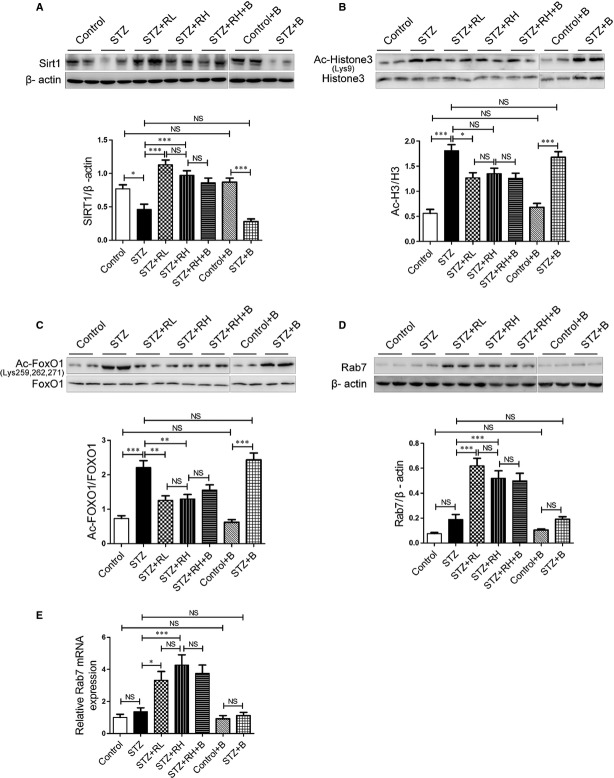
Resveratrol activates sirtuin 1 (SIRT1) and enhances Rab7 expression in diabetic mouse hearts. Western blot analysis of SIRT1 (**A**), acetylated histone 3 (Ac-H3) and total histone 3 (H3) (**B**), acetylated FOXO1 (Ac-FOXO1) and total FOXO1 (**C**) and Rab7 (**D**) in mouse groups. (**E**) RT-PCR analysis of Rab7 mRNA expression. Data are mean ± SEM. *n* = 4. *P < 0.05, **P < 0.01, ***P < 0.001.

The protein expression of SIRT1 significantly decreased in diabetic hearts, which was increased by resveratrol treatment after 16-week treatment (Fig. 4A). SIRT1 expression was not affected by bafilomycin A1 treatment (Fig. 4A).

Substrates of SIRT1 include histone and FOXO proteins [21]. Thus, we evaluated SIRT1 activity with the protein levels of acetylated histone 3 (Ac-H3) and acetylated FOXO1 (Ac-FOXO1). The protein levels of Ac-H3 and Ac-FOXO1 were increased in diabetic hearts and decreased after resveratrol treatment for 16 weeks (Fig. 4B and C) and therefore long-term resveratrol treatment may enhance the deacetylase activity of SIRT1 in diabetic hearts. Bafilomycin A1 treatment did not alter the acetylated level of histone 3 or FOXO1 (Fig. 4B and C).

Both the mRNA and protein levels of Rab7 were increased in diabetic mouse hearts with resveratrol treatment (Fig. 4D and E). Moreover, bafilomycin A1 did not affect the mRNA and protein levels of Rab7 (Fig. 4D and E). Thus, resveratrol stimulated the activity of SIRT1 and the expression of Rab7 in diabetic hearts, with no effect of inhibited autophagy flux.

### H_2_O_2_-induced oxidative stress impairs autophagic flux and decreases SIRT1 activity in H9C2 cells

**Fig. 5:**
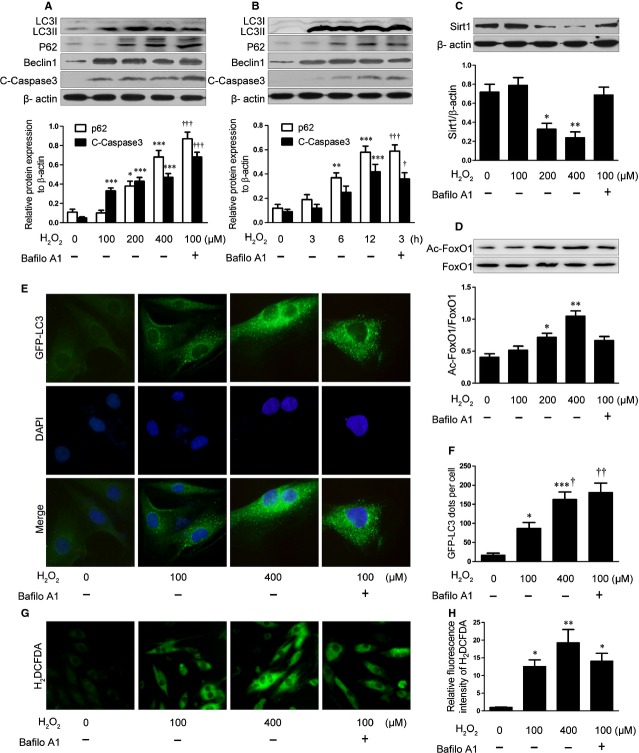
H_2_O_2_-induced oxidative stress impairs autophagic flux and decreases SIRT1 activity in H9C2 cells. H9C2 cells were treated with concentrations of H_2_O_2_ for 12 hrs with or without bafilomycin A1 (Bafilo A1; 100 nM). Western blot analysis of protein levels of p62, cleaved caspase 3 (C-Caspase3) (**A**), SIRT1 (**C**), Ac-FOXO1, and total FOXO1 (**D**). Data are mean ± SEM. *P < 0.05, **P < 0.01, ***P < 0.001 *versus* cells without H_2_O_2_ and bafilomycin A1; †††*P* < 0.001 *versus* cells with H_2_O_2_ (100 μM) without bafilomycin A1. (**B**) H9C2 cells were treated with H_2_O_2_ (400 μM) for different times with or without bafilomycin A1 (100 nM). Western blot analysis of protein levels of p62 and C-Caspase3. Data are mean ± SEM. **P < 0.01, ***P < 0.001 *versus* cells without H_2_O_2_ and bafilomycin A1; †*P* < 0.05, †††*P* < 0.001 *versus* cells with H_2_O_2_ (3 hrs) without bafilomycin A1. (**E**) Fluorescent microscopy of H9C2 cells transfected with GFP-LC3 lentivirus, then exposed to 100 or 400 μM H_2_O_2_ for 12 hrs with or without bafilomycin A1 (100 nM). (**F**) Quantification of GFP-LC3 dots in cells. Data are mean ± SEM. *P < 0.05, ***P < 0.001 *versus* cells without H_2_O_2_ and bafilomycin A1; †*P* < 0.05, ††*P* < 0.01 *versus* cells with H_2_O_2_ (100 μM) without bafilomycin A1. (G) Fluorescent microscopy of H9C2 cells stained with H_2_DCFDA after exposed to 100 or 400 μM H_2_O_2_ for 12 hrs with or without bafilomycin A1 (100 nM). (**H**) Relative fluorescence intensity of H_2_DCFDA in H9C2 cells. Data are mean ± SEM. *P < 0.05, **P < 0.01 *versus* cells without H_2_O_2_ and bafilomycin A1.

To investigate whether oxidative stress alters autophagic flux *in vitro*, we incubated H9C2 cells, a cardiomyoblast cell line from rat, with H_2_O_2_. The ratio of LC3II to LC3I and protein level of p62 were up-regulated with increased concentration and prolonged performance time of H_2_O_2_ as were levels of beclin1 and cleaved caspase 3 (Fig. 5A and B). Bafilomycin A1 further increased p62 and cleaved caspase 3 expression (Fig. 5A and B). H_2_O_2_ treatment decreased the protein level of SIRT1 and increased Ac-FOXO1 level at 200 and 400 μM for 12 hrs (Fig. 5C and D).

Immunofluorescence analysis demonstrated a significant accumulation of GFP-LC3 dots in H9C2 cells with 400 or 100 μM H_2_O_2_ and bafilomycin A1 for 12 hrs (Fig. 5E and F). Intracellular oxidant production was assessed by measuring fluorescence intensity of H_2_DCFDA. 100 or 400 μM H_2_O_2_ treatment up-regulated the ROS level in cells (Fig. 5G and H). Redundant or overactive oxidative stress may inhibit autophagic flux and SIRT1 activity and increase apoptosis in H9C2 cells.

### SIRT1 is essential for resveratrol amelioration of H_2_O_2_-impaired autophagic flux in H9C2 cells

**Fig. 6:**
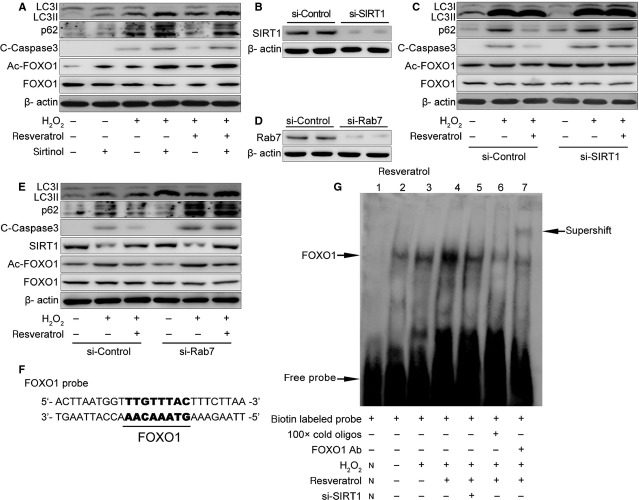
Resveratrol ameliorates autophagic flux in H9C2 cells under oxidative stress *via* SIRT1 and Rab7. (**A**) Western blot analysis of autophagy and apoptosis-related proteins in H9C2 cells treated with or without H_2_O_2_ (400 μM), resveratrol (25 μM), and sirtinol (25 μM) for 12 hrs. (**B**) Western blot analysis of Sirt1 and β-actin in H9C2 cells transfected with Sirt1 or non-targeting siRNA. (**C**) Western blot analysis of autophagy and apoptosis-related proteins in H9C2 cells with or without H_2_O_2_ (400 μM) and resveratrol (25 μM) for 12 hrs after transfection. (**D**) Western blot analysis of Rab7 and β-actin in H9C2 cells transfected with Rab7 or non-targeting siRNA. (**E**) Western blot analysis of SIRT1 and autophagy and apoptosis-related proteins in H9C2 cells with or without H_2_O_2_ (400 μM) and resveratrol (25 μM) for 12 hrs after transfection. (**F**) Nucleotide sequences of probes used in the EMSA analysis. (**G**) H9C2 cells were pre-treated with control or SIRT1 siRNA, then with or without H_2_O_2_ (400 μM) and resveratrol (25 μM) for 12 hrs. Nuclear extracts were analysed by EMSA. Lane 1: negative control without nuclear extract; lanes 2, 3, 4, and 5: nuclear extract from H9C2 cells; lane 6: competition for binding by the unlabeled probe; lane 7: nuclear extract pre-treated with anti-FOXO1 antibody for supershift analysis.

To investigate whether resveratrol ameliorating impaired autophagic flux in cells with oxidative stress depends on SIRT1, we used sirtinol to inhibit SIRT1 activity [22]. Resveratrol decreased the levels of p62, cleaved caspase 3 and Ac-FOXO1 under oxidative stress with H_2_O_2_ in H9C2 cells, and these effects were reversed by sirtinol (Fig. 6A).

To further establish that the observed effects were because of SIRT1 inhibition, we knocked down SIRT1 with siRNA. SIRT1 protein was significantly decreased in H9C2 cells after transfection with SIRT1 siRNA (Fig. 6B). Similar to sirtinol, SIRT1 siRNA abolished the effects of resveratrol on the expression of p62, cleaved caspase 3 and Ac-FOXO1 under oxidative stress with H_2_O_2_ (Fig. 6C). Therefore, the activity and expression of Sirt1 are necessary for resveratrol amelioration of impaired autophagic flux and apoptosis under oxidative stress in H9C2 cells.

### Rab7 is required for resveratrol amelioration of H_2_O_2_-impaired autophagic flux in H9C2 cells

To evaluate whether Rab7 is required in resveratrol-enhanced autophagic flux under oxidative stress *in vitro*, H9C2 cells were transfected with Rab7 siRNA before resveratrol and H_2_O_2_ treatment. Rab7 knockdown was confirmed by western blot analysis (Fig. 6D). The levels of p62 and cleaved caspase 3 were higher with Rab7 than control siRNA knockdown after H_2_O_2_ treatment (Fig. 6E). Furthermore, resveratrol-decreased p62 and cleaved caspase 3 levels were attenuated by Rab7 knockdown (Fig. 6E). However, Rab7 knockdown did not affect the protein levels of SIRT1 or Ac-FOXO1 (Fig. 6E). Stimulation of autophagic flux by resveratrol under oxidative stress in H9C2 cells may depend on Rab7 expression.

### Resveratrol enhanced the DNA-binding ability of FOXO1 through SIRT1

To investigate whether resveratrol increases the transcriptional activity of FOXO1, we designed a probe containing the binding site of FOXO1 in accordance with the promoter region of Rab7 (Fig. 6F) and detected the DNA-binding activity of FOXO1 in nuclear extracts from H9C2 cells by EMSA. The binding activity of FOXO1 was increased with resveratrol treatment and significantly decreased with Sirt1 siRNA transfection (Fig. 6G). In addition, we observed a supershift in expression with anti-FOXO1 antibody (Fig. 6G), which confirmed that the binding was FOXO1-specific. Resveratrol may increase the DNA-binding ability of FOXO1 to the promoter region of Rab7 through Sirt1-dependent pathway.

## Discussion

The present study demonstrates that resveratrol improves cardiac dysfunction and remodelling and attenuates cardiomyocyte apoptosis and oxidative stress injury, associated with regulation of autophagic flux, in diabetic mice. Resveratrol increases the activity of SIRT1 and the expression of Rab7 in diabetic mouse hearts. SIRT1 activity and Rab7 expression are required for resveratrol amelioration of impaired autophagic flux in H9C2 cells under oxidative stress. Furthermore, SIRT1 is necessary for resveratrol-increased DNA binding of FOXO1 to the promoter region of Rab7.

Diabetic hearts show accumulation of autophagosomes [23]. However, whether this accumulation is associated with the redundant formation or inhibited degradation, or both, is unclear. In the present study, we found accumulation of autophagosomes in hearts of STZ-induced diabetic mice, as well as up-regulated levels of LC3II, beclin1 and p62, which agree with findings by Mellor *et al*. [24]. Beclin1 binds the class III phosphatidylinositol-3 kinase (PtdIns3KC3) complex, which is required for PtdIns3KC3-dependent autophagosome formation [25]. The protein p62 is a link between LC3 and ubiquitinated substrates and is degraded in autolysosomes [26]. Thus, up-regulated level of beclin1 is associated with increased autophagosome formation, and p62 accumulation is associated with autophagic degradation dysfunction. Collectively, these observations suggest impaired autophagic flux in the diabetic heart. However, these findings in the STZ-induced diabetic mice are not consistent with those in OVE26 mice [27], which may be attributed to the different genetic backgrounds of the mice.

Impaired autophagic flux leads to decreased degradation of misfolded proteins or damaged organelles, such as depolarized mitochondria, which would result in cardiac hypertrophy, fibrosis, apoptosis and heart failure eventually [28]. Cardiac dysfunction of diabetic mice is aggravated by autophagic flux inhibited with a lysosomal inhibitor, bafilomycin A1. However, bafilomycin A1 treatment has no significant effect on cardiac function of control mice. These results indicate deferent dependency level of autophagic flux between diabetic and non-diabetic mice.

The protective effects of resveratrol on diabetic cardiomyopathy have been considered related to its beneficial actions on energy metabolism, mitochondrial function and manganese superoxide dismutase activity [29]. In the present study, the beneficial effects of resveratrol on diabetic hearts were associated with its regulation of autophagic flux. Resveratrol decreased p62 expression in diabetic hearts, but had no significant effect on beclin1 level. Furthermore, inhibition of autophagic degradation with bafilomycin A1 attenuated almost all the beneficial effects of resveratrol on diabetic cardiomyopathy. Resveratrol may improve the degradation, but not formation, of autophagosomes in diabetic hearts. The dysfunction of autophagosomes degrading promotes cardiomyocytes apoptosis in ischemia/reperfusion injury [19]. In the present study, resveratrol reduced the ratio of apoptosis in diabetic heart, and bafilomycin A1 reversed it, which was detected by TUNEL assay. These results indicate that resveratrol inhibits apoptosis probably through improving autophagic flux. However, TUNEL has the limitation on apoptosis detection. TUNEL detects not only DNA fragmentation but also single-stranded DNA breaks with free 3′-OH terminals. Therefore, TUNEL labels necrotic as well as apoptotic cells, and even living cells with increasing activity of DNA repair [30].

No significant dose-dependent effects of resveratrol were observed in the present study. Actually, a very low-dose resveratrol (2.5 mg/kg/day) was found enough to induce autophagy in rodent hearts [13]. Thus, two dose of resveratrol (60 and 300 mg/kg/day) used here might produce similar effects on diabetic hearts.

Decreased activity of SIRT1 and impaired autophagic function can be observed simultaneously in age-related disease with redundant oxidative stress [31]. Whether SIRT1 regulates autophagy in diabetes remains elusive. In agreement with others [12], we found that SIRT1 expression was significantly decreased in diabetic hearts. Moreover, resveratrol up-regulated the protein level of SIRT1 as well as its deacetylase activity. Genetic knockdown or inhibition of SIRT1 activity abolished the improved autophagic flux with resveratrol under oxidative stress *in vitro*. Thus, these results suggest that SIRT1 is necessary for resveratrol-induced enhancement of autophagic flux.

FOXO transcription factors are at the interface of crucial cellular processes, orchestrating programmes of gene expression that regulate apoptosis and autophagy. SIRT1 deacetylates FOXO1 and positively regulates FOXO1-dependent gene transcription [32]. In this study, we found high-acetylated FOXO1 expression in diabetic hearts and in cultured cells with exogenous oxidative stress, which could be decreased by resveratrol. Besides SIRT1, class I and class II histone deacetylases (HDACs) may also induce deacetylation of FOXO1 or histone 3 [33]. Our results do not exclude the involvement of other HDACs-dependent deacetylation of FOXO1 in modulation of autophagic flux. Nevertheless, EMSA results suggested our hypothesis that resveratrol increases the transcriptional activity of FOXO1 through SIRT1. A previous study showed that SIRT1 could deacetylate FOXO3 in response to oxidative stress and stimulate the expression of autophagy-related proteins [34]. The roles of other FOXO members in resveratrol-induced improvement of autophagic flux need to be further researched.

Rab7 is a crucial factor in the maturation of autophagosomes and their fusion with lysosomes [35]. Up-regulated Rab7 was observed in STZ-induced painful diabetic neuropathy [36]. In the present study, we found Rab7 mRNA and protein expression stimulated by resveratrol in diabetic mouse hearts. Furthermore, Rab7 knockdown abolished resveratrol amelioration of impaired autophagic flux *in vitro*. These data suggest that Rab7 is required in resveratrol alleviation of dysfunctional autophagic flux. Because deacetylated FOXO1 increases the expression of Rab7 in starvation-induced autophagy [37], resveratrol may enhance the expression of Rab7 under oxidative stress by stimulating the transcriptional activity of FOXO1, which was confirmed by EMSA. Thus, the SIRT1/FOXO1/Rab7 axis is stimulated by resveratrol.

These results provide novel insights into the role of impaired autophagic flux in diabetic cardiomyopathy. Resveratrol improves cardiac dysfunction and remodelling and attenuates cardiomyocyte apoptosis and oxidative stress injury in diabetic mice, associated with the regulation of autophagic flux through SIRT1/FOXO1/Rab7 axis, which suggests a therapeutic strategy for diabetic cardiomyopathy.

## Supporting information

**Data S1** Details of animal protocol, echocardiography, TEM, cellular ROS detection and EMSA. **Table S1** Mouse primers used for real-time RT-PCR. **Table S2** General characteristics of mouse groups. **Table S3** Echocardiographic parameters for mouse groups.
