# Supplementary material for: Resveratrol-enhanced autophagic flux ameliorates myocardial oxidative stress injury in diabetic mice
Source: J Cell Mol Med. 2014 Jun 1;18(8):1599–611. doi: 10.1111/jcmm.12312 (PMC4190906; doi:10.1111/jcmm.12312)
Supplement: Supplementary file 1 [file jcmm0018-1599-SD1.docx]

**Supporting information**

1) Supplementary methods: Details of animal protocol, echocardiography, TEM, cellular ROS detection and EMSA.

2) Supplementary Table 1: Mouse primers used for real-time RT-PCR.

3) Supplementary Table 2: General characteristics of mouse groups.

4) Supplementary Table 3: Echocardiographic parameters for mouse groups.
